# Umbilical Artery Access for PDA Stenting: Feasibility in Select Anatomy

**DOI:** 10.1007/s00246-026-04215-1

**Published:** 2026-03-26

**Authors:** Thomas Roberts, Marjan Hesari, Danica Peterson, Juliana Gomez-Arostegui, Clinton Fulk, Justin Ryan, Denise Suttner, John Nigro, Howaida El-Said

**Affiliations:** 1 Department of Pediatrics, University of California, San Diego, La Jolla, San Diego, CA USA; 2 Division of Cardiology, Rady Children’s Hospital, San Diego, CA USA; 3 Helen and Will Webster Foundation 3D Innovations Lab, Rady Children’s Hospital, San Diego, CA USA; 4 Department of Neurological Surgery, UC San Diego Health, La Jolla, CA USA; 5 Department of Neonatology Surgery, UC San Diego Health, La Jolla, CA USA; 6 Division of Cardiovascular and Thoracic Surgery, Department of Surgery, University of California, San Diego, CA USA

**Keywords:** Patent ductus arteriosus stenting, Umbilical artery access, Congenital heart disease, Neonate, Minimally invasive intervention

## Abstract

Patent ductus arteriosus (PDA) stenting is a less invasive alternative to surgical shunts in ductal-dependent congenital heart disease. Umbilical artery access for PDA stenting offers femoral-like retrograde advantages while avoiding associated risks, however is underreported in this context. This study evaluates umbilical artery access feasibility and outcomes for PDA stenting. A case series included seven neonates undergoing PDA stenting via umbilical artery access between 01/17/2020 and 10/30/2024 at Rady Children’s Hospital, San Diego. Patient demographics, PDA morphology, and procedural details were collected. Existing umbilical arterial lines were rewired and exchanged for a 4 F Flexor sheath (Cook Medical Bloomington, Indiana, USA), advanced through the iliac artery to the descending aorta. A coaxial system was used to cross the PDA and deploy coronary stents with a wire-locking technique for controlled positioning. Post-procedure, patients received dual antiplatelet therapy with the umbilical catheter remaining in situ until stability. This study (71% female) had a median gestational age of 38 weeks and a median birth weight of 2.97 kg. The median age at catheterization was 8 days (range: 3–10). PDA morphology originated from the descending aorta (*n* = 4, 57.1%) and the brachiocephalic artery (*n* = 3, 42.9%). The median procedure time was 126 min (range: 42–161 min), with a median fluoroscopy time of 24.7 min (11.4–58.6). No intra- or post-procedural complications occurred. Median stent diameter was 3.5 mm; 85.7% required one stent. Scheduled reinterventions, as part of staged palliation, were performed in a subset of patients. Umbilical artery access for PDA stenting is a safe and effective alternative in select cases, with no acute complications in our study.

**Supplementary Information:**

The online version contains supplementary material available at 10.1007/s00246-026-04215-1.

## Introduction

Patent ductus arteriosus (PDA) stenting has emerged as a minimally invasive alternative for maintaining ductal patency in neonates with ductal-dependent congenital heart disease (CHD) [1]. However, complications including vascular injury, stent thrombosis, ductal spasm, and in-stent stenosis necessitate careful access-site selection and procedural planning [2, 3].

Established access routes to the PDA include the femoral artery (FA), carotid artery (CA), and axillary artery (AA) [3]. Femoral access is preferred for PDA’s that arise from the descending aorta, whereas the carotid and axillary arteries are more suitable for those arising from the undersurface of the arch [3, 4]. Each access option carries inherent risks: femoral access is known to be susceptible to vessel spasm and thrombosis [5], while accessing the carotid and axillary arteries can cause pseudoaneurysms [6]. Furthermore, though recent systematic studies praise the axillary approach for complex anatomy, it is acknowledged that it carries a technical demand and risk profile, including thrombosis and dissection, even under ultrasound guidance [7].

Given the aforementioned access risks and complex anatomic variants of the PDA, alternative access options warrant exploration. We aim to share our experience of using the umbilical artery to access the PDA for stenting. As a non-essential vessel that undergoes postnatal closure, the umbilical artery offers a brief neonatal window for intervention without long-term vascular consequences. This case series describes the technical approach, suitable anatomy, and outcomes of umbilical artery access for PDA stenting.

## Methods

We conducted a case series at Rady Children’s Hospital, San Diego, where all patients who underwent a PDA stenting procedure for pulmonary ductal-dependent CHD were collected. The stenting procedures took place between 01/17/2020 and 10/30/2024. Patients were only deemed candidates for the umbilical approach if they had viable umbilical access and suitable PDA anatomy. If either criterion was not met, the patient was planned for an alternative access route (femoral, axillary, or carotid) and thus excluded from this study. As such, all seven patients described in this study had successful UAC access.

Baseline patient demographics, procedural data including stent specifications and fluoroscopy parameters, and follow-up data were collected and analyzed using median values given the limited cohort size (*n* = 7). To define PDA morphology, Qureshi’s tortuosity classification was used [8].

### Umbilical access methodology

**Fig. 1:**
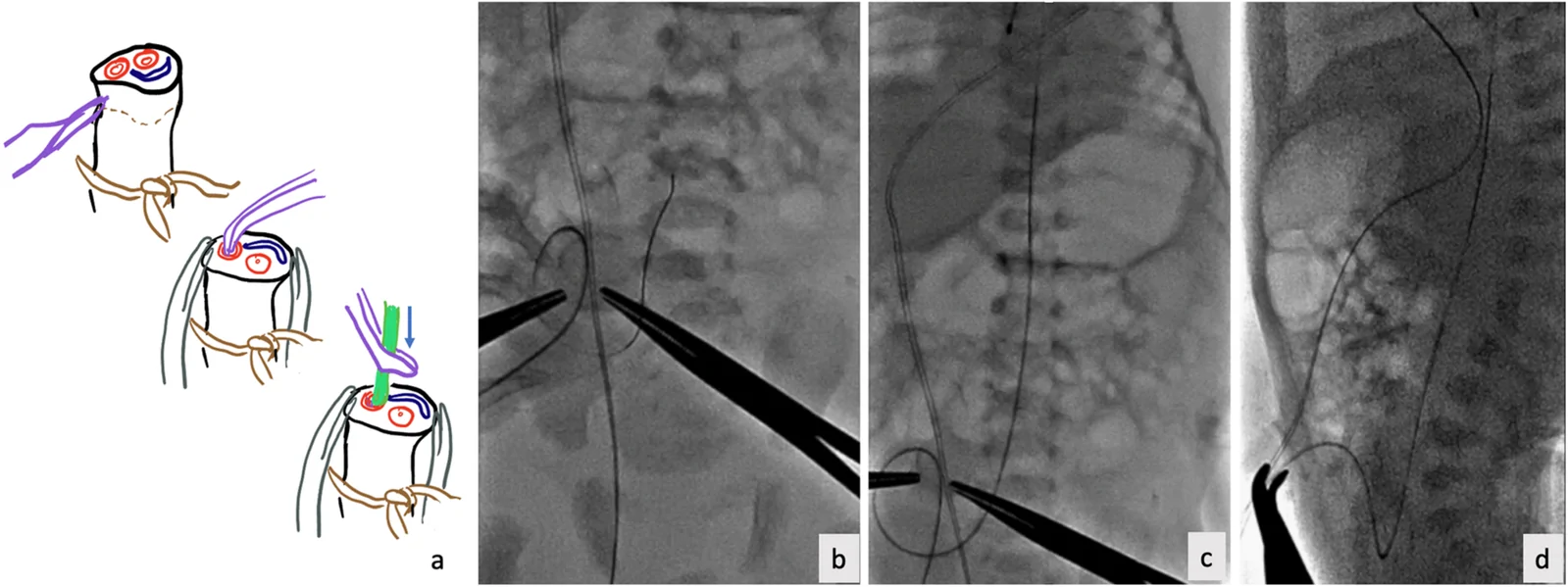
**a** Demonstrates the steps to access the umbilical artery. An umbilical tape is tied to the lower part of the stump, the stump is cut, then the artery is identified and dilated with forceps then the catheter is inserted vertically as shown. **b** Shows artery forceps holding the stump up with BMW wire manipulated through the UAC. **c**, **d** Advancing the wire into the aorta in antero-posterior (AP) and lateral view, - note the down and up trajectory on AP view (**c**) and lateral view in (**d**) (See video 1)

All procedures are performed via rewiring an existing umbilical arterial line (UAC) placed by neonatology in the Neonatal Intensive Care Unit (NICU) (Fig. 1). Whilst a formal cut-off delay after birth for this access method currently doesn’t exist, the viability of this approach is largely attributed to umbilical stump viability, with pathology affecting the umbilicus (infection, thrombosed umbilical arteries, abdominal wall defects) and natural stump desiccation (typically after 7 days) being the primary drivers for umbilical access disqualification. Once an arterial line is in place, provided integrity is maintained, the procedure can exceed the typical time for stump desiccation (7days) as seen within our cohort (8-day median age at catheterization), though this should be assessed on a case-by-case basis. At our institution, umbilical venous and arterial catheters are routinely placed in all neonates with congenital heart disease and maintained for up to two weeks. De novo UAC placement for PDA stenting may be considered if the umbilical stump remains viable, with the highest success within 24–48 h of life.

Appropriate pre-procedural 3D mapping from CT scans is conducted for each patient (as demonstrated in Fig. 3a). All procedures were performed in the cardiac catheterization laboratory under fluoroscopic guidance. Prior to the procedure, patients had UACs placed by neonatology in the NICU without routine systemic anticoagulation, as per institutional protocol. At the time of catheterization, intravenous heparin is administered at 100 units/kg with a target activated clotting time (ACT) of approximately 250 s. Routine antibiotic prophylaxis is not administered while the UAC remains in place; however, prophylactic antibiotics are given at the time of line exchange in the cardiac catheterization laboratory.

**Fig. 2:**
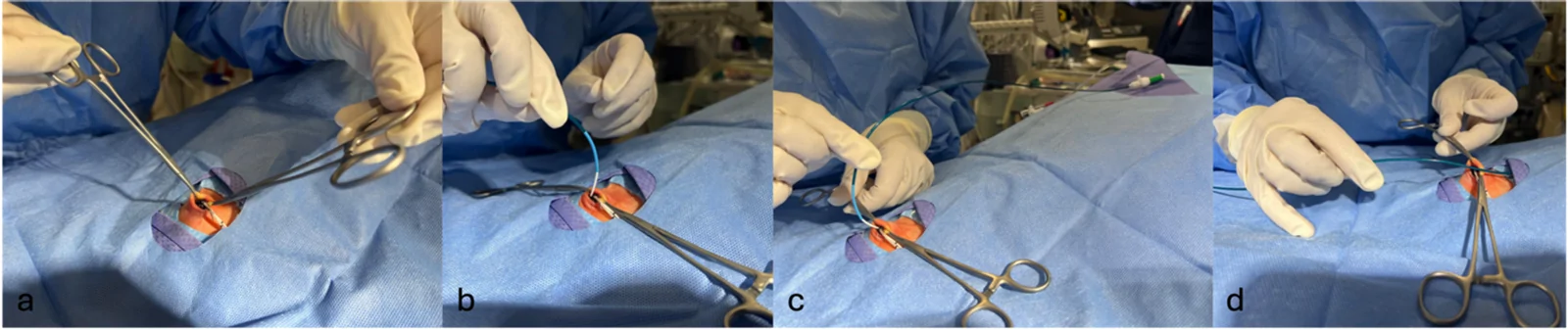
**a** Illustrates the artery forceps being pulled upward during manipulation. **b** Demonstrates the initial insertion of the 4 French Flexor sheath. **c** Shows the 4 French Flexor sheath in its initial cranial orientation. **d** Shows the same 4 French Flexor sheath after it has been turned into the caudal position. (See video 2)

**Fig. 3:**
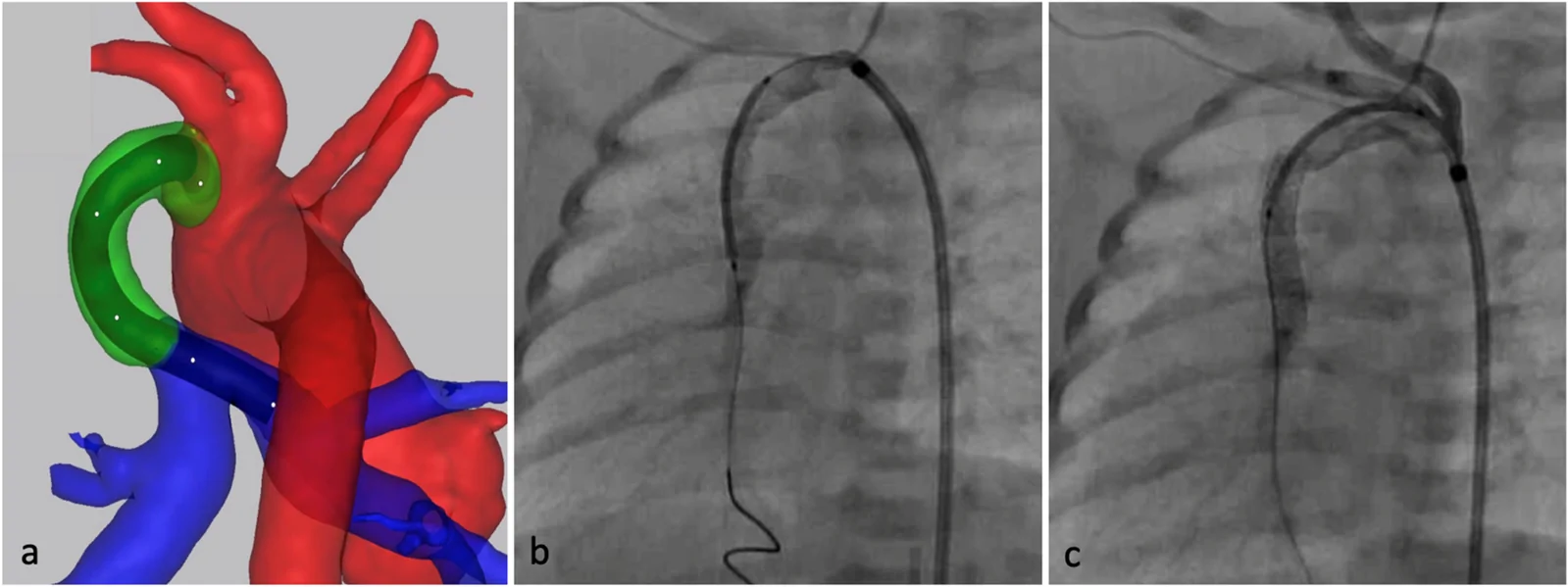
Shows PDA stent placement through the 4 F long sheath placed through the umbilical artery. **a** Displays 3D segmentation from a CT scan for pre-procedure planning - note the computer-generated cylinder in the PDA to simulate placement. **b** Wire across the PDA in the distal right lower pulmonary artery branch twisted and locked in place with the distal stent advanced to the distal PDA, with **c** showing a second stent placed to cover the entire PDA length after angiography following initial stent deployment (See video 3)

**Fig. 4:**
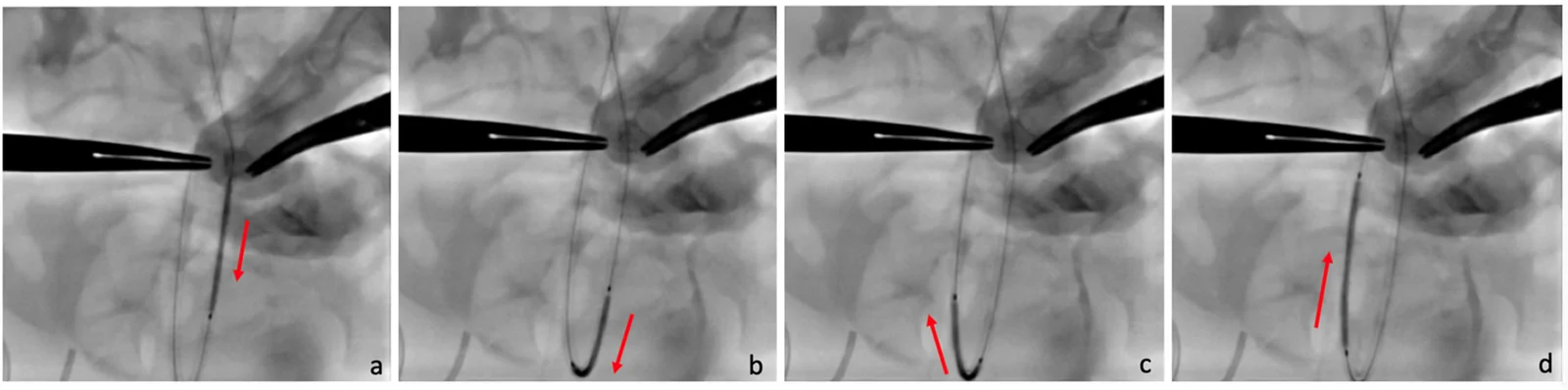
Shows the PDA stent progressing through the long sheath. Note the stent trajectory: First, descending caudally in **a**, **b**, before making the turn in **c** and then ascending cranially in **d** (See video 3)

1. Two hemostats are placed on either side of the umbilical stump to help lift it during the procedure (Fig. 2) (Video 1).
2. A 0.021 wire (Cordis Corporation, Miami Lakes, Florida, USA) is placed in the UAC, the UAC is removed, and a 4 F 60 cm Angled glide catheter (Terumo, Somerset, New Jersey, USA) is placed over the wire. Note that it is very important during any catheter manipulation to gently lift the stump upward to accommodate the natural downward and upward curvature of the umbilical artery as it transitions into the iliac artery, facilitating catheter advancement (Fig. 2). The 0.021 wire is exchanged for a 0.035 wire (Cordis Corporation, Miami Lakes, Florida, USA) and a pre-curved 4 F 45 cm Flexor sheath (Cook Medical, Bloomington, Indiana, USA) is inserted over the 0.035 wire. This wire exchange is done to avoid any mismatch between the 0.021” wire and the 0.035” inner lumen of the sheath dilator. Again, it is imperative to lift the umbilical stump during sheath advancement. To facilitate insertion, the sheath is first advanced from the cranial position, following the natural curve of the vessel to navigate the iliac artery. After successfully making this turn and reaching the isthmus, the sheath is then flipped caudally. Technical steps up to flexor sheath placement are displayed in video 2.
3. The sheath is parked in the descending aorta close to the aortic end of the PDA (Fig. 3b). Heparin 100 u/kg is given to maintain an ACT of 250. A coaxial system consisting of a 0.014” × 300 cm BMW wire (Abbott, Santa Clara, California, USA) and a Renegade microcatheter (Boston Scientific, Marlborough, Massachusetts, USA) inside a 4 F angled glide catheter is prepped and placed through the long sheath. The angled glide is placed at the mouth/aortic end of the PDA – note that it is important not to advance the angled glide across the PDA. The BMW wire and Renegade microcatheter are manipulated very carefully and gently across the PDA to avoid ductal spasm, dissection or thrombosis.
4. The BMW wire and Renegade microcatheter are then advanced to the desired branch pulmonary artery, and the wire is twisted clockwise for 5–6 turns to lock in the lung parenchyma [9]. This allows dynamic wire tension adjustment resulting in controlled stent positioning, with increased tension improving trackability and reduced tension enabling conformation to ductal anatomy [16]. Post-deployment angiography is performed to confirm optimal stent placement and whether another overlapping stent is needed to cover the aortic end. The path of the stent through the long sheath to the PDA is shown in Fig. 4. Once the stent is in place, the Renegade catheter is advanced back over the twisted/locked wire and unlocked (counterclockwise rotation) and removed through the Renegade micro catheter. A final angiogram is performed through the sheath with the Renegade micro catheter in place to make sure no additional stents are needed after slight repositioning of the stent after wire removal. An example of post-deployment angiography is shown in Fig. 3b, with a further stent utilized in Fig. 3c to cover the entire PDA length.
5. Post stent placement the 4 F 45 cm Flexor sheath is exchanged over a 0.021 wire for a 5 F double lumen UAC catheter.
6. A loading dose of Plavix 2 mg/kg is given via the naso-gastric tube prior to leaving the catheterization lab. The patient is returned to the Cardiothoracic Intensive Care Unit (CTICU) with the UAC catheter in place and started on daily aspirin (20.25 mg, equivalent to ¼ of an 81 mg baby aspirin tablet) and Plavix 1 mg/kg/day.
7. There is no need for flat time. The patient can be extubated in the catheterization laboratory or shortly after returning to the unit. The UAC remains in place until the patient is stable, typically for a few days. To remove it, an umbilical tape is tied around the stump and tightened to control bleeding.

## Results

In total, seven patients had a PDA stent placed through the umbilical artery, with a female predominance (*n* = 5, 71.4%) over males (*n* = 2, 28.6%). Regarding patient demographics, the median gestational age was 38 weeks (range: 34 + 3 to 39 + 4 weeks) with a median birth weight of 2.97 kg (range: 2.02–3.83 kg). Full patient demographics and cardiac pathology profiles can be found in Table 1.

**Table 1:** Clinical and demographic characteristics of umbilical access study (*n*=7)

Patient No.	Sex	Gestational Age (Weeks+Days)	Birth Weight (Kg)	Diagnosis	Co-morbidities	Age at PDA stent (Days)	Weight at Cath. (Kg)	Fluoroscopy Time at PDA stent	Procedure time min	No. of PDA Stents	Tortuosity Type (I/II/III)	PDA Origin	Arch Side
1	M	39w+4	3.83	PA/IVS	N/A	3	3.8	0:38:50	130*	1	Type II	Descending aorta	Left arch
2	M	39w+2	2.74	TOF like DORV	N/A	7	2.8	0:58:34	161	3	Type III	Left brachiocephalic artery	Right arch
3	F	36w+6	2.98	TGA, PA/VSD	N/A	9	3	0:21:00	102**	1	Type I	Descending aorta	Left arch
4	F	38w+2	3.41	PA/IVS	N/A	3	3.31	0:11:26	42	1	Type I	Descending aorta	Left arch
5	F	39w+4	2.97	PA/IVS	Laryngeal cleft repair	10	3.33	0:12:26	50	1	Type I	Descending aorta	Left arch
6	F	34w+3	2.58	CAVC, PA, Infradiaphragmatic TAPVR, Heterotaxy	Prematurity	8	2.71	0:24:44	126#	1	Type II	Right brachiocephalic artery	Left arch
7	F	37w+2	2.02	DILV, normally related great arteries with hypoplastic TV and RV with restrictive bulboventricular foramen	N/A	8	2.27	0:34:23	138**	1	Type III	Left brachiocephalic artery	Right arch

*IVS* Intact ventricular septum, *TOF-like DORV* Double outlet right ventricle with a tetralogy of Fallot-like anatomy, *TGA* Transposition of great arteries, *VSD* Ventricular septal defect, *CAVC* Complete atrioventricular canal defect, *PA* Pulmonary atresia, *TAPVR* Total anomalous pulmonary venous return, *DILV* Double inlet left ventricle, *PS* Pulmonary stenosis, *PDA* Patent ductus arteriosus

Additional procedures performed during the same intervention: * RF perforation and pulmonary valvuloplasty, ** Atrial septostomy and # Vertical vein stent

PDA tortuosity was Type I in three patients (42.9%), Type II in two patients (28.6%), and Type III in two patients (28.6%). The PDA originated from the descending aorta in 4 patients (57.1%) and from a brachiocephalic artery in three patients (42.9%). The aortic arch was left-sided in five patients (71.4%) and right-sided in two patients (28.6%).

**Fig. 5:**
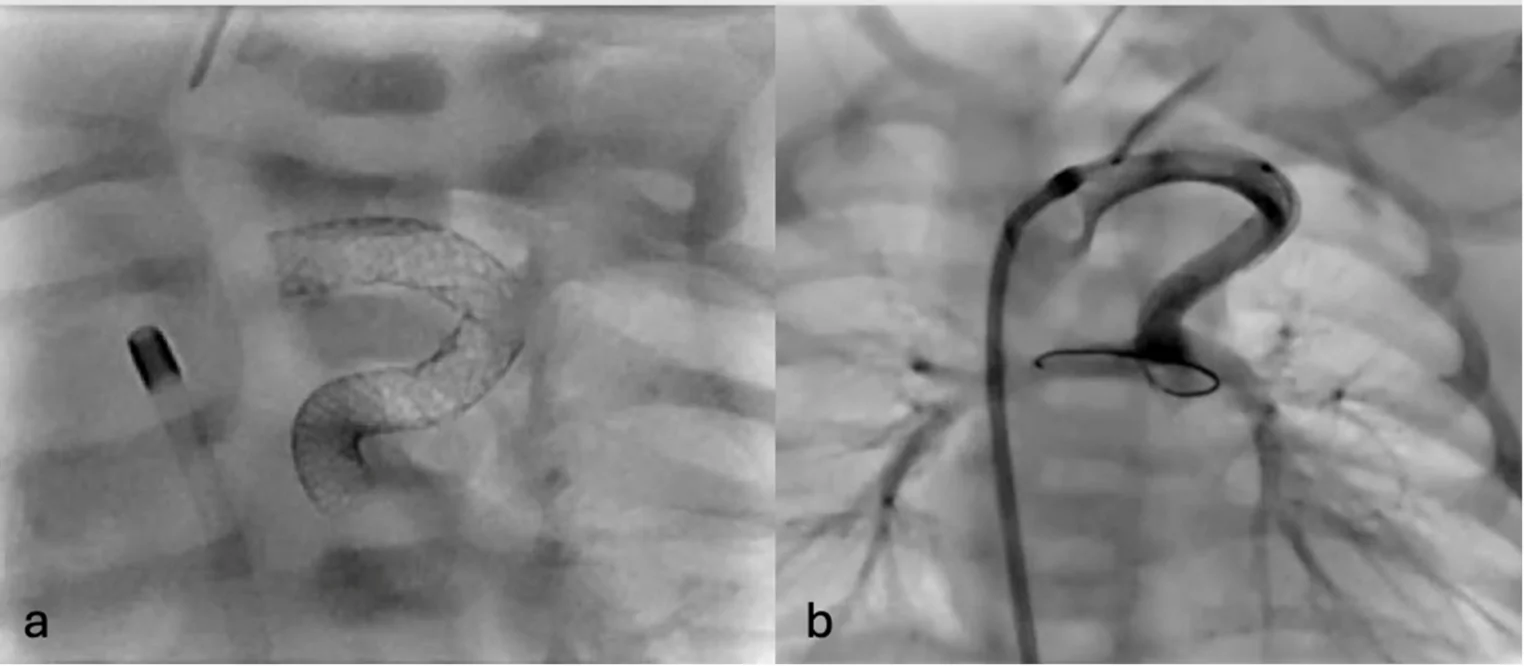
This figure shows 3-D reconstructions of the aortic arch and PDA anatomy. **a** Illustrates a patient with two PDAs - one arising from the undersurface of the arch and another from the brachiocephalic trunk. **b** Shows a PDA originating from the descending aorta. Solid arrows indicate PDA types suitable for umbilical arterial stenting (brachiocephalic and descending aorta). The dashed arrow marks the PDA arising from the undersurface of the arch, which should be approached via the carotid or axillary artery rather than the umbilical route

The median age at catheterization was 8 days (range: 3–10 days). The median procedure time was 126 min (range: 42–161 min), with a fluoroscopy time of 24 min (range: 11.43–58.57 min) and radiation dose of 108.42 cGy·cm² (range: 63.37–275.70 cGy·cm²). There were no recorded intra-interventional events (Fig. 5).

**Fig. 6:**
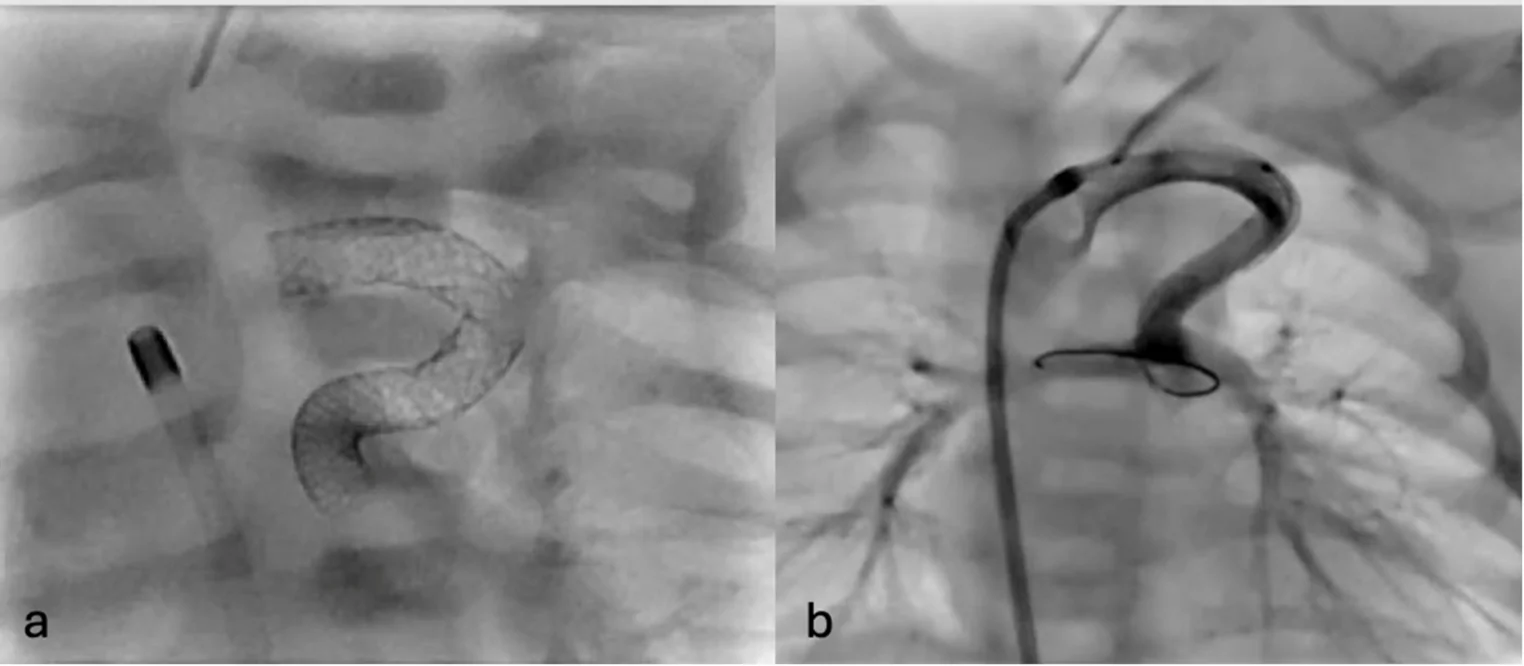
**a** showing three 3.5-mm stents to fully cover a long Type III PDA arising from the innominate artery. Initial two stents left an unstented gap, requiring a third stent for complete coverage. **b** demonstrates the final fluoroscopy imagine of the 3 stents in situ. This represents the only case in the cohort requiring multiple stents (See video 4)

Stent diameter was primarily 3.5 mm (85.7%, *n* = 6), with one patient requiring a 2.5 mm diameter stent. Median length was 20 mm (range: 12–30 mm). In most cases a single stent was used (85.7%, *n* = 6). A demonstration of stent placement shown in video 3. One patient required three stents; although an anomaly, this case involved a complex type III PDA morphology arising from the left brachiocephalic artery, necessitating three stents to cover the entirety of the PDA. Two stents were initially used in this case; however, further imaging demonstrated an unstented area between the two, thus a third was placed (Video 4). Fluoroscopy images shown in Fig. 6a, b capture the tri-stent nature of this case. Full interventional details and PDA morphology across the cohort can be found in Table 2.

**Table 2:** Procedural characteristics of initial PDA stent placement in umbilical access cohort (*n*=7)

Parameter	Value
Age at First Catheterization (days)	8 (3–10)
Weight at first initial Catheterization (kg)	3.05 (2.75–3.32)
*PDA Origin (n=7)*	
Descending Aorta	4 (57.1%)
Brachiocephalic trunk	3 (42.9%)
*PDA Type (n=7)*	
Type III	*2 (28.6%)*
Type II	*2 (28.6%)*
Type I	*3 (42.9%)*
*Number of Stents*	
Single Stent	*6 (85.7%)*
Triple Stent	*1 (14.3%)*
Stent Diameter (mm)	3.5 (2.5–3.5.5.5)
Stent Length (mm)	20 (18–30)
Procedure Time (minute)	126 (42–161)
Fluoroscopy Time (minute)	24.73 (11.43–58.57)
Radiation Dose (cGycm^2^)	108.42 (63.37–275.70.37.70)

There were no intraprocedural or immediate post-procedural complications such as bleeding, thrombosis, or stent migration. Post-procedural assessment included standard clinical examination with femoral pulse documentation as a screening measure for vascular complications. Re-intervention of the PDA was required in two patients:

- Patient 4 underwent PDA stent balloon angioplasty and pulmonary valvuloplasty at 3 months, followed by a further main pulmonary artery (MPA) stent and PDA balloon dilation at 4 months.
- Patient 7 underwent balloon angioplasty of the PDA stent and pulmonary artery at 3 months.
- These reinterventions were part of staged palliation and not for the treatment of acute stent failure.

## Discussion

Traditionally, surgical shunts were used for cyanotic CHD, but interventional methods like PDA stenting are becoming a preferred alternative in suitable cases due to evolving technology and evidence [10, 11]. Studies have shown reduced complications (OR 0.4, *P* = 0.006) and improved pulmonary artery growth with PDA stenting [12]. Access site selection is crucial for the success of the procedure.

Access via the FA is most frequently utilized and often preferred due to its larger caliber and ease of ability to achieve safe homeostasis, especially in the neonate population [13]. Although widely adopted, the FA has risks of thrombosis and vessel spasm [5, 14], with some studies suggesting true thrombosis prevalence as high as 23.4% [15]. FA access often necessitates the use of long introducer sheaths for better control and simultaneous injections for positioning [16]. This is a significant consideration, as the use of long sheaths has been identified as an independent risk factor for arterial thrombosis; one study reported a complication rate of 38% compared to 15% with short, matching-size sheaths [16]. Recent developments, such as the wire twisting technique, as described by Kato et al., has allowed the use of the FA regardless of anatomical variations [14]. The umbilical approach shares this retrograde path to the PDA.

Despite the risks associated with FA use, it is still often favored since effective utilization of the carotid and axillary arteries requires both experienced interventionalists paired with careful vascular ultrasound guidance [17]. Even then, the possibility of pseudoaneurysm (a complication reported at 11% in a cohort study by Choudry et al. [17]) or aortic dissection/thrombosis (complications reported for axillary access at 5.6% for both in a cohort study by Haddad et al. [23]) may outweigh the advantage of a shorter catheter time offered by axillary/carotid access in tortuous PDA variants compared with femoral access [14]. However, CA/AA catheterization may be chosen in long, tortuous ductal anatomy (Type II and III [8]) due to favorable co-axial alignment, which facilitates stent delivery and reduces the risk of ductal spasm or malposition [2, 18]. Further to this, recent procedural series highlight the utility of axillary access within duct-dependent pulmonary circulation with tortuous ductal anatomy, as it provides a stable, coaxial approach that is not an end-artery, thereby preserving distal limb perfusion during the procedure [22].

Although the umbilical artery has no postnatal clinical significance, UAC still carries inherent risks. The most frequent complication is catheter rupture, which may cause hemorrhage, particularly if near the umbilical stump [19]. More severe but rarer complications, such as non-occlusive aortic thrombus, have been reported in studies like Levit et al., though this risk correlates with prolonged UAC dwell time and therefore may not apply to short-term interventional use [19].

While literature on umbilical artery access for interventions remains limited, potential procedural risks could theoretically include iliac artery injury or aortic dissection, given the catheter’s trajectory. However, none of these complications occurred in our small cohort. Our umbilical artery access cohort demonstrated excellent procedural safety, with no immediate complications (e.g., bleeding, thrombosis, or stent migration). These findings are mirrored by a recent series by Bazil et al. [20], where umbilical arterial access was used in the context of neonatal neurointervention. This recent series demonstrated a similar safety and efficacy profile of transumbilical access for the endovascular embolization of high-flow cerebrovascular malformations in 21 neonates, with a high technical success rate (20/21 intended cases) and absence of access-specific complications [20]. Although procedural nuances differ, (for example, Bazil et al. preserve their sheath for up to 10 days post-procedure [20]) the foundation that umbilical access is a viable alternative access method remains.

This contrasts with the CA access group (*n* = 20) in Alsawah et al.’s study, which reported a 15% complication rate (primarily pseudoaneurysm and procedural failure) [21]. The FA access cohort (*n* = 20) in that same study had significantly higher complications (45%, *p* = 0.038), including hematoma, thrombosis, and access failure, aligning with known risks in neonates [21]. The FA subgroup within Alsawah et al.’s [21] cohort exhibited comparable PDA typing to our umbilical cohort (60% type I/II); however, their CA population had notedly more complex PDAs (55% type III), limiting the conclusions we can draw from this subgroup.

Umbilical access required a longer median procedure time (126 min) compared to previous CA (71.5 min) and FA studies (75 min) [21, 22]. However, this difference is partly attributable to case complexity within our cohort, with three patients requiring an additional procedure alongside PDA stenting. Despite the longer duration, umbilical access offers important safety advantages, avoiding the neurologic complications associated with carotid or axillary routes (e.g., pseudoaneurysm, hemiparesis [17]) and the vascular complications reported with FA access in neonates [5, 14]. The umbilical approach may also benefit from emerging technologies – such as steerable microcatheters, which have demonstrated efficacy in complex vessel navigation [23]. While the additive effect of steerable microcatheters and umbilical access may produce superior operator experience and patient outcomes, our experience in this study suggests conventional catheterization technology is capable of excellent results in the context of umbilical access.

While umbilical artery access may offer advantages for PDA stenting based on complications, patient selection depends critically on ductal anatomy. This approach is most feasible when the PDA arises from the descending aorta or if it arises from the brachiocephalic trunk allowing straightforward antegrade cannulation. Figure 5 provides a 3D model of this anatomical criterion, showing a bilateral PDA case with one morphology suitable for umbilical access (solid arrow) and another that is not (dashed arrow). As such, pre-procedural imaging is essential to evaluate PDA morphology and determine candidacy for PDA stenting via the umbilical artery route.

It is important to note that umbilical artery access shares the same retrograde trajectory as femoral access and does not offer inherent geometric advantages in navigating PDA tortuosity. Rather, its primary value lies in providing an alternative access site during the brief neonatal window before umbilical closure, potentially preserving femoral vessels for future procedures.

## Limitations

While our study provides promising initial results for umbilical artery catheterization, it is important to address some limitations within the study that may limit wider generalizability. Firstly, although a reasonable variation in PDA subtype was observed amongst our population, our small sample size limits the statistical power we can derive from the analysis. Furthermore, all procedures in our cohort were performed by a single interventionalist and the outcomes achieved may not be easily generalizable across institutions. Finally, the retrospective nature and lack of a control group preclude direct comparison with other access routes; however, the short follow-up period limits assessment of long-term vascular complications or patency outcomes. Additionally, while clinical assessment with femoral pulse examination was performed post-procedure, we did not employ standardized imaging protocols (such as routine vascular ultrasound) to systematically assess for iliac artery patency or aortic injury. Implementation of standardized post-procedural vascular imaging would strengthen future evaluation of this access route and allow for more sensitive detection of subclinical vascular complications.

## Conclusion

This study highlights the feasibility and safety of umbilical artery access as an underutilized approach in select patients in which the PDA arises either from the descending aorta or the brachiocephalic trunk. We saw umbilical artery access for PDA stenting to be a safe and effective alternative in select cases, with no acute complications in our cohort. Further studies are needed to validate its role amongst larger PDA cohorts and compare long-term outcomes to traditional access routes.

## Supplementary Information

Below is the link to the electronic supplementary material.

Supplementary Material 1

Supplementary Material 2

Supplementary Material 3

Supplementary Material 4

## Data Availability

All data generated or analyzed during this study are included within this article.
